# A Dynamic Wheelchair Armrest for Promoting Arm Exercise and Mobility After Stroke

**DOI:** 10.1109/TNSRE.2022.3187755

**Published:** 2022-07-14

**Authors:** Marti Comellas, Vicky Chan, Daniel K. Zondervan, David J. Reinkensmeyer

**Affiliations:** Department of Mechanical and Aerospace Engineering, University of California Irvine, Irvine, CA 92617 USA; Rehabilitation Services, University of California Irvine Medical Center, Orange, CA 92868 USA; Flint Rehabilitation Devices, Irvine, CA 92614 USA; Department of Anatomy and Neurobiology, the Department of Mechanical and Aerospace Engineering, and the Department of Biomedical Engineering, University of California Irvine, Irvine, CA 92617 USA

**Keywords:** Armrest, biomechanics, ergonomics, manual wheelchair, stroke rehabilitation

## Abstract

Arm movement recovery after stroke can improve with sufficient exercise. However, rehabilitation therapy sessions are typically not enough. To address the need for effective methods of increasing arm exercise outside therapy sessions we developed a novel armrest, called Boost. It easily attaches to a standard manual wheelchair just like a conventional armrest and enables users to exercise their arm in a linear forward-back motion. This paper provides a detailed design description of Boost, the biomechanical analysis method to evaluate the joint torques required to operate it, and the results of pilot testing with five stroke patients. Biomechanics results show the required shoulder flexion and elbow extension torques range from −25% to +36% of the torques required to propel a standard pushrim wheelchair, depending on the direction of applied force. In pilot testing, all five participants were able to exercise the arm with Boost in stationary mode (with lower physical demand). Three achieved overground ambulation (with higher physical demand) exceeding 2 m/s after 2–5 practice trials; two of these could not propel their wheelchair with the pushrim. This simple to use, dynamic armrest provides people with hemiparesis a way to access repetitive arm exercise outside of therapy sessions, independently right in their wheelchair. Significantly, Boost removes the requirements to reach, grip, and release the pushrim to propel a wheelchair, an action many individuals with stroke cannot complete.

## I. Introduction

One in six people will have a stroke and over half will incur chronic upper extremity impairment [1], [2]. Repetitive arm movement practice can substantially increase motor recovery [3], yet most individuals do not perform enough movement practice [4], [5] especially in the critical window early after stroke when plasticity is heightened [6], [7]. Approximately 70% of stroke inpatients (and nearly all of those with severe impairments) spend several hours each day sitting passively in manual wheelchairs, sometimes with their paretic arm statically strapped into a static arm trough [8], [9]. Further, when they ambulate in their chair, they are either pushed or taught to self-propel with their “good” arm and leg, further contributing to disuse of the hemiparetic arm.

The goal of this work is to give patients and therapists a novel tool that allows them to collaborate in generating plasticity-inducing levels of arm motor drive outside of formal treatment. The most common current approach to encouraging arm exercise outside of therapy time is to prescribe exercises using a paper handout. Adherence is low with this approach [10]–[13]. As a potential alternative, a large number of sensor- and robot-based arm exercise systems have also been developed. These technologies are comparable in effectiveness to conventional therapy [14], [15], but they are not typically used outside of formal treatment times as they require patients to move to a gym where the equipment is set up or to be transferred into a device. Other studies showed that stroke survivors (able to propel a wheelchair) who engaged in extra physical activity self-ambulating obtained a better functional recovery [16]. The approach we describe here is aimed at making appropriate arm exercise more readily accessible by integrating it with the wheelchair that stroke patients often spend time sitting passively in.

The device described here takes into account our experience developing and testing lever-drive wheelchairs for stroke rehabilitation [17]–[19]. We found that individuals with severe arm impairment in the chronic stage of stroke retain sufficient strength and coordination with their paretic arm to manoeuvre bimanual, lever-driven wheelchairs [17], [18]. Participants with stroke exhibited largely healthy biomechanics, with minimal shoulder hiking/leaning or trunk inclination. Their arm muscle EMG patterns were similar to those used by unimpaired participants, with such practice activating elbow extensor and should flexor muscles, a prime target for arm movement training after stroke.

We modified this initial lever drive chair design with a hand clutching design to allow turning in place and backing up [17]. In a randomized control trial, we found that exercise with this device in the subacute phase of stroke led to a reduction in arm impairment compared to conventional treatment, demonstrating the therapeutic potential of wheelchair-based arm exercise after stroke [18]. However, therapists noted that the hand clutching technique required for overground propulsion placed a high cognitive demand on patients, and most struggled to learn to use the device for propulsion. They exercised mainly in a stationary mode with the overground drive disengaged. Therapists stated they would be more likely to use the device if it were smaller, could be quickly attached to a patient’s conventional manual wheelchair, did not impede normal use of the wheelchair, and aided in mobility. Satisfying these requirements while achieving the desired therapeutic arm exercise presented a design challenge.

The solution presented in this paper, called Boost, is a new type of wheelchair armrest that quickly clicks into a wheelchair frame just like a conventional armrest. Thus, the user does not need to transfer to a special wheelchair to use the device. Yet, like our previous designs, Boost allows users to activate arm muscles in a way that is appropriate for the early stages of stroke recovery, via supported elbow extension and shoulder flexion. We first describe the design of Boost. We then provide a biomechanical analysis of its operation. Finally, we describe results and therapist feedback from preliminary testing of Boost with five stroke inpatients.

## II. Materials and Methods

### A. Hardware Design

**Fig. 1.**
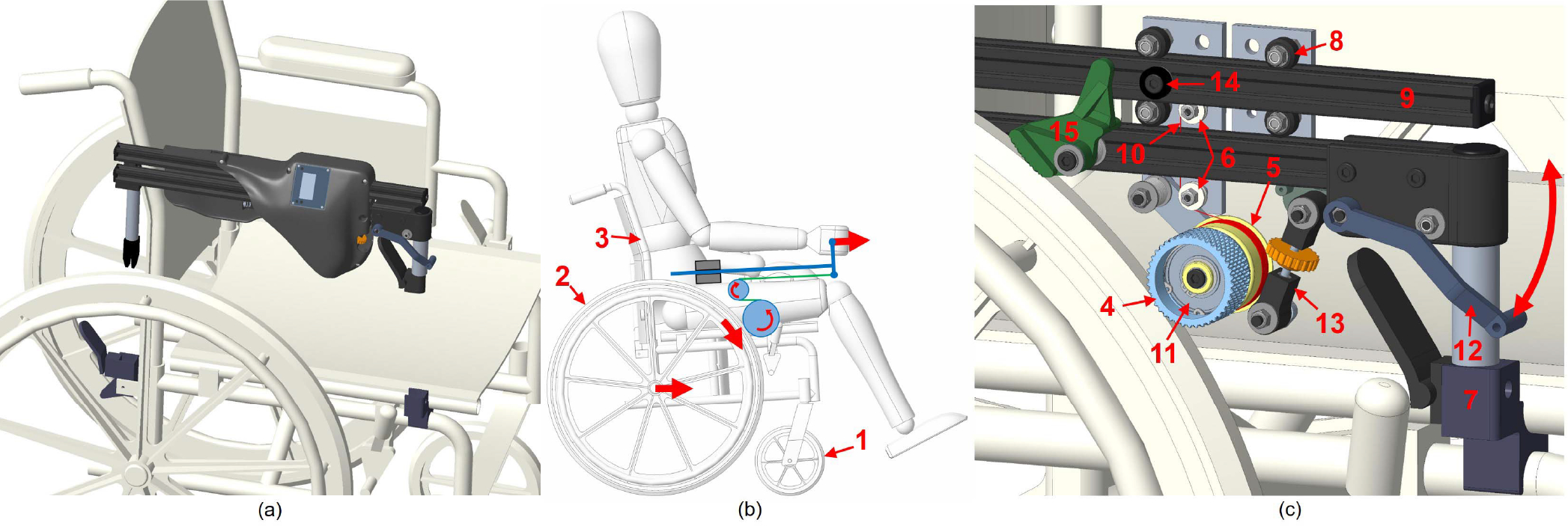
(a) Boost shown just before clicking into a wheelchair. (b) Conceptual operating principle. A linear slide parallel to the armrest guides forward/backward motion of the hand. A cable attached to the handle passes around a pulley then wraps around a friction drive that can be engaged to propel the wheelchair. (c) Transmission mechanism. See text for detailed description.

Fig. 1 left shows how Boost clicks into a standard wheelchair, replacing the arm rest. Boost allows users to practice a forward/backward linear arm reaching motion in two modes: 1) with the wheelchair remaining stationary (Stationary Mode), or, 2) with the wheelchair being propelled by the linear arm motion (Overground Mode). Fig. 1 middle shows the operating principle. The hand is guided by a linear slide parallel to the armrest. A cable attached to the handle passes around a pulley then wraps around a friction drive that can be engaged to propel the wheelchair.

**Fig. 2.**
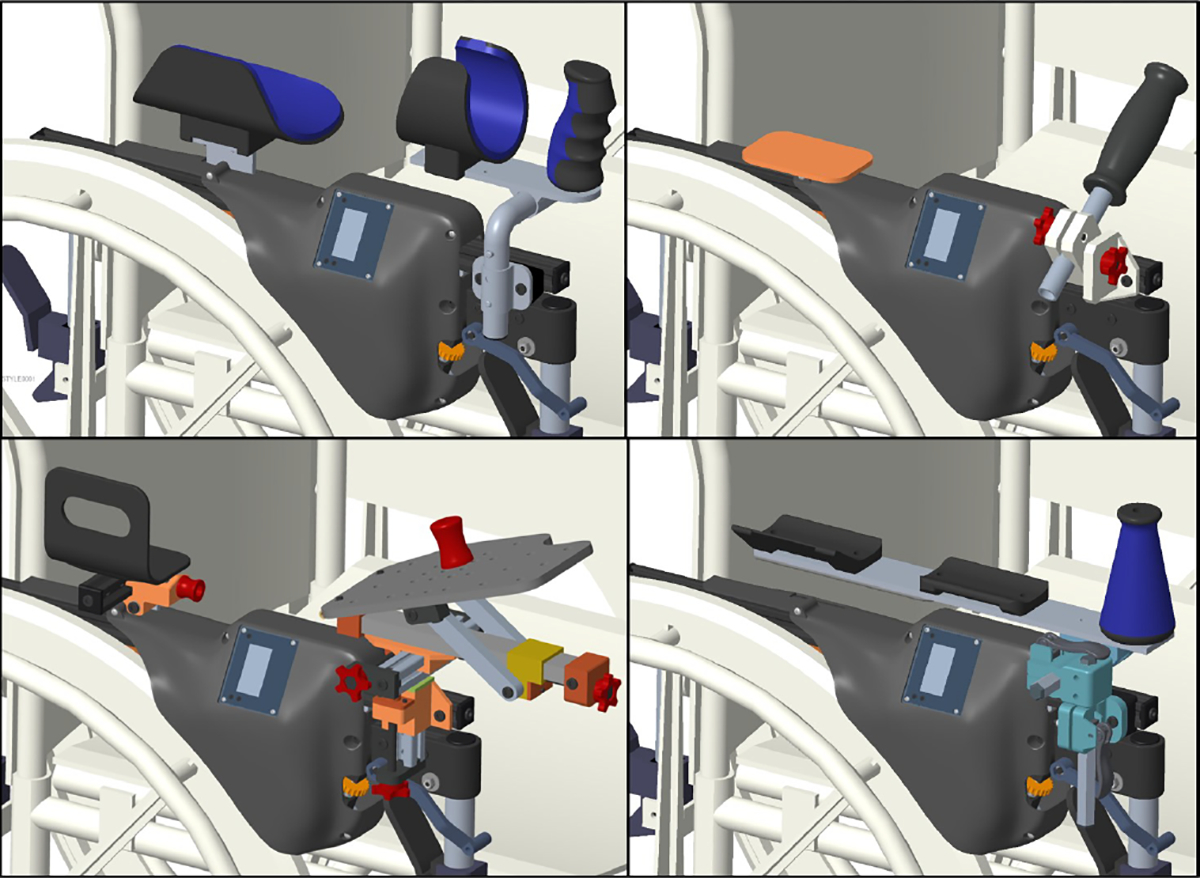
Options for hand and forearm support. Top Left: A static elbow support to prevent shoulder abduction with a dynamic forearm/wrist support and ergonomic handle with two degrees of freedom of adjustment to promote beneficial propulsion mechanics; Top Right: A simple handgrip with two degrees of freedom of adjustment that can be used by individuals with milder impairments; Bottom Left: A flexible hand support plate with three degrees of freedom and a “peg-board” design for modular placement of custom finger and wrist posts to allow the user to adjust the vertical, horizontal, and rotational position of the hand; Bottom Right: A two degrees of freedom adjustment with full forearm (elbow to wrist) articulated support that provides support against gravity for individuals with more severe impairments.

Fig. 1 right shows the detailed design of the transmission system. Four ball bearing wheels (8) guide the movement of the rail (9). The cable (10) is fixed to an anchor point at the front of the rail, where an adjustable hand support can be attached (not shown in Fig. 1, see Fig. 2). The cable, hidden inside the rail slot, is guided by pulleys (6). The other end of the cable is wrapped around the friction drive, which is comprised of a reel (5) that is coupled to a friction disk (4) via a one-way bearing (11). A spiral spring embedded in the reel keeps the tension of the cable regardless of mode, rail position, or phase of propulsion. The spring keeps the slide in its starting position when it is not being actuated by the arm, and slightly assists the user in returning their arm to its initial position (i.e. it assists elbow flexion).

The main axle of the transmission system is attached to the armrest frame (parallel to the wheelchair wheel’s axis) by a four-bar linkage with two limiting positions. A mechanical lever (12), easily accessible with a user’s opposite hand (i.e. a hemiparetic patient’s “good” hand), allows the user to switch between these two limiting positions, switching between the Stationary and Overground operating modes. In Stationary Mode, the friction disk is disengaged from the wheelchair wheel, allowing the user to push the slidable rail back and forth against the reel spring with their arm without causing movement of the wheelchair. In Overground Mode, the friction disk constantly contacts the wheelchair wheel. When the user pushes the slidable rail forward (propulsion phase), the linear force is transferred into a torque on the wheelchair wheel by friction and the user contributes to propelling the wheelchair with their arm. When the user moves the arm backward, no force is transferred to the wheelchair’s wheel due to the one-way-bearing. Using an accessible hand nut, the length of the four-bar linkage’s coupler bar (13) can be adjusted to modify the pressure of the friction disk against the wheelchair wheel in overground mode. This allows Boost to be adapted for multiple wheelchair geometries and reduces slippage between the friction disk and the wheelchair wheel.

In addition to assisting with propulsion, Boost allows a user to pull the slide backward at the end of its range of motion to activate a friction brake, which is useful for descending ramps or steering. A brake stopper (14) is attached to the slidable rail (Fig. 1 right). A brake pad (15) acting through a lever mechanism is attached to the frame such that it can provide a braking friction force on the perimeter of the wheelchair wheel when the brake stopper is in contact due to the pullback force provided by the user. The lever mechanism is spring loaded so that the brake pad is kept separated from the perimeter of the wheel when no pullback force is applied. In Overground Mode, the spring-loaded brake is automatically engaged when the wheelchair rolls backward due to the one-way bearing. Thus, Boost provides an automatic, self-locking capability, as the brake prevents the wheelchair from rolling backwards when pulling the arm back for another push while ascending ramps. To ambulate backwards with Boost, a user must switch the drive to Stationary mode (an operation that takes less than one second) and use their feet or the pushrims to propel the chair.

Boost is designed to attach to most standard commercial wheelchairs using two modular anchor points, just like conventional wheelchair armrests. Specifically, since armrest anchor specifications are not standardised across commercial wheelchair manufacturers, Boost is designed to support a library of anchor points—each designed for a specific wheelchair manufacturer—that can be quickly swapped out for simple installation. The current anchor points described here are designed to work with a specific commercial wheelchair [20]. The Boost armrest “U” shape frame can be attached and detached from the wheelchair by a standard “click” system that uses the same anchorage parts as the original armrest (see Fig. 1 right, item 7). Up to two Boost devices can be simultaneously attached to one wheelchair, replacing each armrest. Notably, when attached to a wheelchair, Boost’s integrated, low-profile design does not interfere with conventional pushrim use.

As an individual patient’s size and physical capabilities will vary widely across Boost’s intended user base, we developed a variety of multi-adjustable attachments options for hand and elbow support. Here, our goal was to allow clinicians and patients to optimize the comfort, safety, and ergonomics of the hand and elbow supports by providing a large range of adjustability options with different degrees of freedom (Fig. 2). Every support was designed to attach to Boost’s slidable rail. Specific adjustment points were included to make arm position during propulsion more appropriate for stroke patients by allowing the shoulder, elbow, and hand joints to move together in the parasagittal plane. This helps to keep the elbow close to the body to avoid shoulder abduction during use, a motion known to limit elbow extension due to an abnormal joint coupling that is common after stroke (the flexion synergy). At the same time, Boost promotes elbow extension and shoulder flexion and avoids torsion of the shoulder, which is desirable as the shoulder girdle muscles are often weakened after stroke [21]. Additionally, no hand grip force is required to use Boost, an important consideration since hand grasp is typically impaired after stroke.

### B. Biomechanical Analysiss

**Fig. 3.**
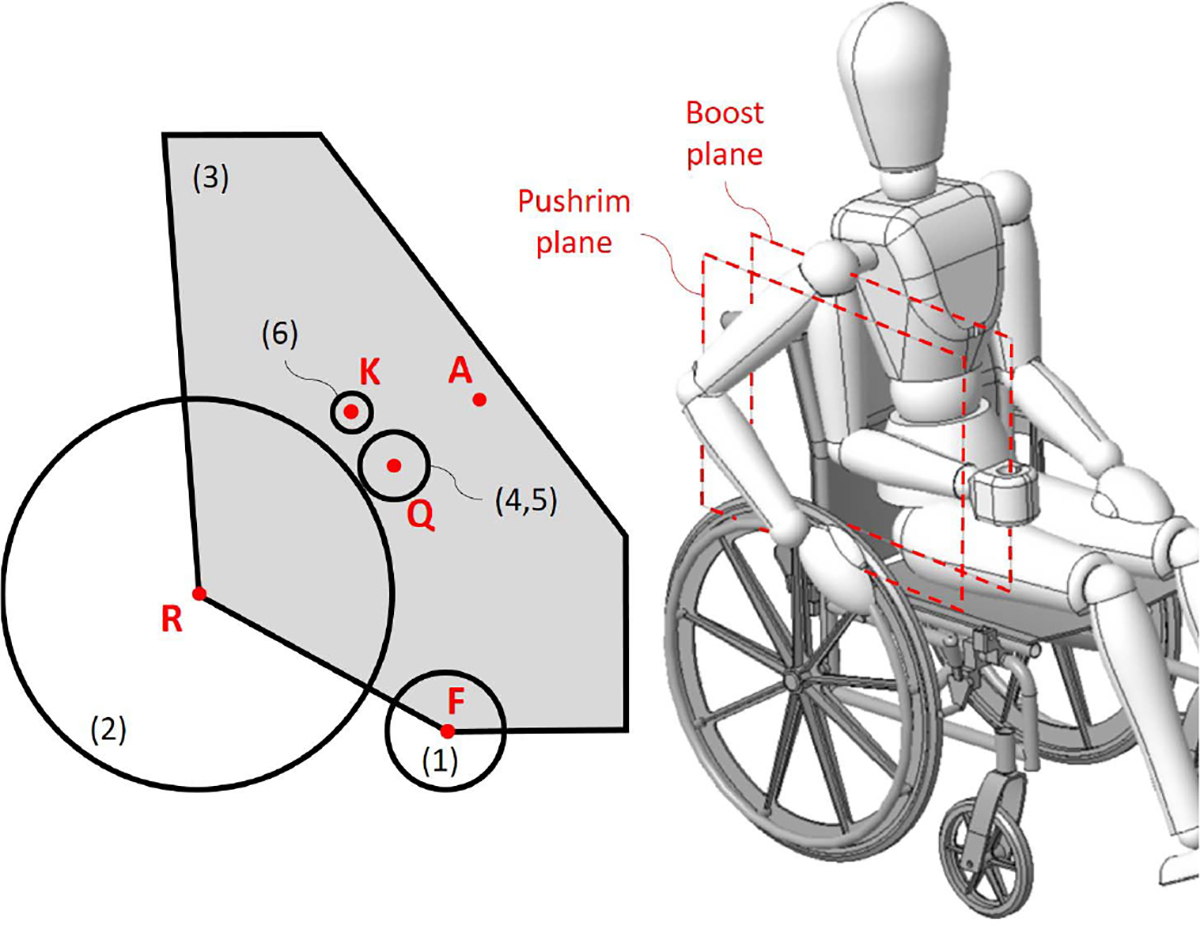
Left: Wheelchair-user 2-D multibody system. Right: Hand movement planes for Boost and the standard pushrim.

We observed from initial experiments with Boost that patients found propelling Boost easier, but we did not know if this could be attributed to reduced joint torque requirements. The aim of the biomechanical analysis was to quantify the range of arm joint torques required to propel a wheelchair using Boost, comparing them to the standard pushrim propulsion method. We modelled the chair and the user as a 2D multibody system, representing half of the whole wheelchair-user system split by the symmetrical plane. This resulted in six bodies for the Boost model (front wheel (1), rear wheel (2), wheelchair frame + user (3), friction disk (4), reel (5) and pulley (6), see Fig. 1-b, c and Fig. 3 left) and three bodies for the standard pushrim model (front (1) and rear (2) wheels and the wheelchair frame + user (3)). Points F, R, K and Q represent the pin points of the bodies with the wheelchair frame, and H represent the alternating position of the hand (i.e. H would be somewhere on the rim when using the standard propulsion method. Additionally, we modelled the arm of the user as a 3-body system (upper arm, forearm and hand) also in the 2D plane (Fig. 5-c). We considered the forearm and the hand to be constantly aligned. In order to reduce the number of degrees of freedom, we considered no torso movement while performing the propelling action. Therefore, the shoulder was assumed to be in a steady position. This assumption is in accordance with previous studies [18] although it still needs experimental validation with Boost. For the standard pushrim model, the upper arm segment length was accordingly shortened taking into account its angle deviation from the plane of study due to shoulder abduction. Fig. 3 right shows the two planes of study for either Boost or the standard pushrim. The two-dimensional characteristic of the model was deemed appropriate since the out-of-plane components of the hand force are substantially smaller than the in-plane components when propelling a regular wheelchair [22]. The out-of-plane force components are aimed to be even smaller when using Boost since the push direction and all the arm joints are in the same plane.

Kinematic and dynamic mathematical models were independently defined. Boost and standard pushrim methods were evaluated separately. Additionally, the wheelchair-user model and the arm model were treated separately. The wheelchair-user kinematic models evaluate displacement (s), velocity (v) and acceleration (a) of the chair by inverse solving (deriving) or forward solving (integrating) its mathematical functions. The arm kinematic models evaluate, by trigonometric relations, the angles (upper arm, forearm and hand) and the coordinate positions (hand, wrist, elbow and shoulder) in a forward or inverse solving. The dynamic models for either the wheelchair-user or the arm were defined combining the general rigid body dynamic equations (Equations 1 and 2) evaluated on the free body diagrams of each of the bodies of the system. Either accelerations or forces can be obtained from depending on if the forward or inverse solving strategy is used.


(1) $$\sum F_{i}=m_{i}\cdot {\ddot{r}}_{G_{i}};\;i=1\ldots n$$

(2) $$\sum {\left(M_{i}\right)}_{z}=J_{G_{i}}\cdot {\overset{..}{\theta }}_{G_{i}};\;i=1\ldots n$$

The rolling friction between the wheelchair wheels and the floor (e = 3.81 · 10^−3^m) and between the wheelchair wheels and the Boost drive system (e_D_ = 3.26 · 10^−3^m) were obtained experimentally. No aerodynamic resistance (i.e. drag) was considered since the velocity of the wheelchair is low. The torque of the spiral spring (M_S_ = 41.2 · 10^−3^N · m) that keeps the tension on the cable of the reel was experimentally found to be constant through Boost’s range of motion. The wheelchair’s dimensions and weights corresponded to an 18” wide commercial wheelchair [20]. The anthropometrics of the user correspond to an 82 kg and 6.2 feet adult male [23], [24]. The contributions of inertia and gravity on the arm were disregarded for this study. The average speed (0.22m/s), the full cycle time (1.5s) and the percentage of propulsion (60%) and recovery (40%) phase were estimated from experimental tests and deemed appropriate for a stroke patient based on observational data. We defined the propulsion phase as the time used by the user propelling the wheelchair and the recovery phase as the time used by the user to return to the starting position of the propulsion.

**Fig. 4.**
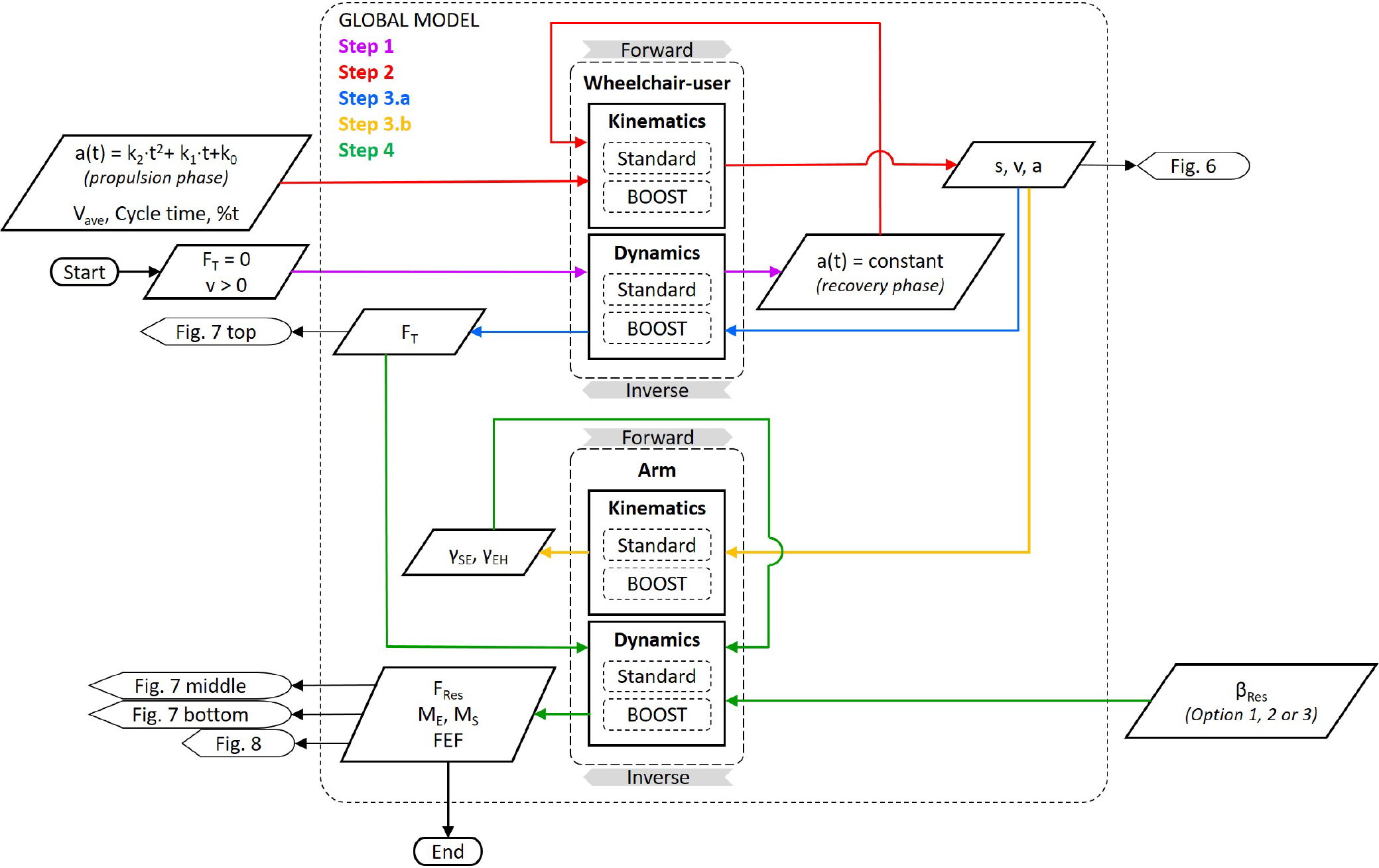
Biomechanical analysis process diagram.

The evaluation of the arm requirements (force and torques) can be described as a 4-step process (Fig. 4).

In a first step, the wheelchair-user dynamic models were solved in a forward analysis to evaluate the constant acceleration during the recovery phase. This analysis was performed considering an instant situation where the wheelchair was moving (velocity is higher than zero) and the user was not generating force (applied force is null).

In a second step, the wheelchair-user kinematic models were solved in a forward analysis to obtain the velocity and position along the whole cycle knowing the constant acceleration value during the recovery phase and assuming that the acceleration during the propulsion phase had a quadratic function shape. Other parameters previously mentioned (average speed, full cycle time and proportion between propulsion and recovery time) were needed to complete this analysis.

**Fig. 5.**
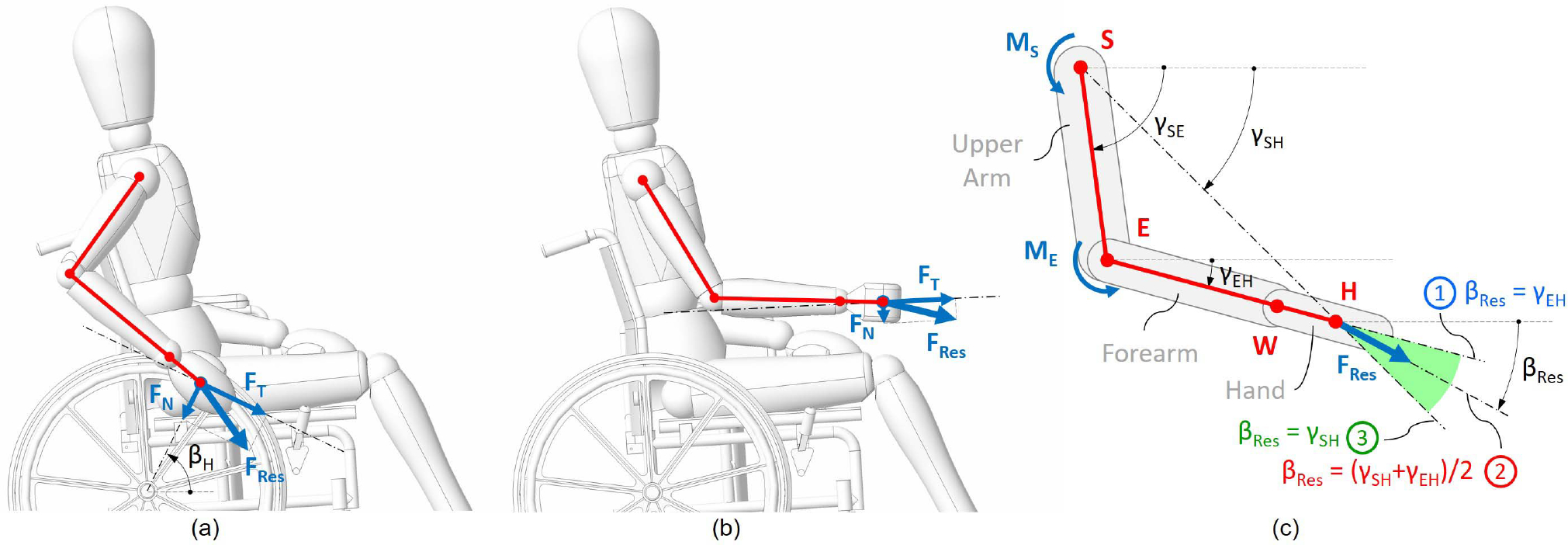
Resultant force (i.e. applied force) vector (F_Res_) and its tangential and normal decomposed vectors (F_T_, F_N_) at the point of application for a standard pushrim wheelchair (a) and for Boost (b). Also in (a): Angle of the axis that passes through the center of the wheel and the hand (*β*_H_). For a generic arm configuration (c): the 3 body members (upper arm, forearm and hand) considered for the 2D arm multibody system. Pin points between bodies: shoulder (S), elbow (E) and wrist (W). Position of the hand (H). Orientation of the bodies referenced to a horizontal plane: Upper arm angle (*γ*_SE_) and forearm and hand angle (*γ*_EH_). Angle of the axis that passes through the shoulder and the hand (*γ*_SH_). Angle of the direction of the resultant force (*β*_Res_) and its 3 possible options analyzed. Shoulder and elbow joint torques (M_S_, M_E_) and resultant force (F_Res_) vectors. Region (green area) between the minimum effort directions for the elbow and the shoulder.

**Fig. 6.**
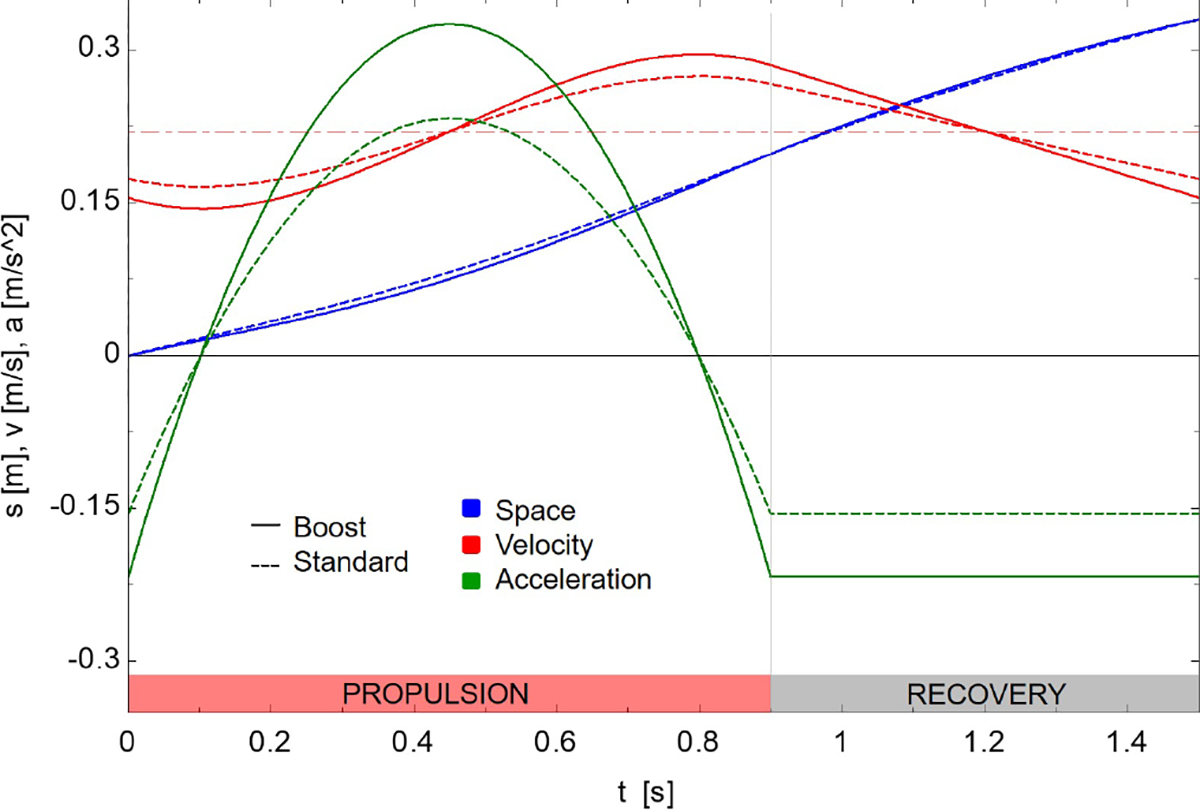
Kinematic performance of the wheelchair.

The acceleration during the propulsion phase was considered to fit a quadratic concave function based on a theoretical tangential force with the same shape. This assumption is in accordance with other studies where the theoretical tangential force was assumed to follow a similar function (for instance, an isosceles triangle function, a fourth degree polynomial function, or a sine function) [25]. Experimental studies (e.g. [26]) found that actual measured forces applied by users also have this kind of bell shape, starting and ending at null values and reaching a peak in between. Once the full kinematic conditions were defined (displacement, velocity and acceleration (see Results Fig. 6)), the wheelchair-user dynamic models were solved in an inverse analysis (step 3.a) to evaluate the ideal force (tangential force) required to propel the wheelchair along the whole cycle. In parallel, also with the kinematic conditions used as input data, the arm kinematic models were solved in an inverse analysis (step 3.b) to evaluate positions and angles of the arm parts. To do so, an initial position of the arm was given. For Boost, the angle between the forearm and the upper arm was assumed to be close to 90°. For the standard pushrim model, the angle of the axis that passes through the contact point of the hand at the pushrim and the center of the wheel with respect to the floor plane (See *β*_H_ on Fig. 5-a) was considered to be 83° at the beginning of the propulsion phase. This is the mean angle that a non-wheelchair user would use to start the propulsion phase according to [27]. The force that the user applies with their hand to propel the wheelchair was modelled as a resultant force decomposed into its tangential and normal components, with the tangential component being the ideal force from the mechanical point of view. Fig. 5-a, b show these forces for Boost and for the standard pushrim models.

In a final stage (step 4), the arm dynamic models were used to evaluate the magnitude of the applied force (resultant force) as well as the required torques at the arm joints and the efficiency of the applied force, evaluated as the ratio between tangential and resultant force, also known as the fraction of effective force (FEF) [35]. To do so, one more assumption needed to be made: the direction of the applied force (resultant force), which depends on how the user performs the propelling action. Many factors have been found to affect the direction of a user’s applied force, including the user’s physical and cognitive conditions, ability, type of chair (seat height, weight distribution, rolling friction), push frequency, and wheelchair speed [28]–[30]. Experimental studies that trained subjects to apply forces in a mechanically more effective direction found that the most effective propulsion technique from a mechanical point of view is not necessarily the most efficient method of propulsion from a biological point of view [31]. Furthermore, [32] also stated that shoulder moments are augmented when propelling with a more efficient force at the pushrim. Given this uncertainty in modelling the applied force, we considered instead a probable range of applied forces. We define this range as the angular range between the two minimum effort directions of the elbow and the shoulder, as defined by [22], which we name as the biomechanically preferred direction range. This is the region where the shoulder performs a flexion movement and the elbow an extension movement, as is common practice during actual wheelchair propulsion [33], [34]; muscle activation patterns used to propel LARA are consistent with propulsion by shoulder flexion and elbow extension [18]. Based on that premise, we decided to evaluate our models across three possible options: two aligned with the minimum effort directions of the elbow and the shoulder and one between these two directions. Fig. 5-c shows the three angles of the force directions analyzed and, as a green region, the area between the two minimum effort directions.

### C. Pilot Testing

Five individuals who had recently suffered a stroke resulting in arm impairment and who were inpatients at an acute rehabilitation unit were asked to use Boost, first in Stationary Mode and then in Overground Mode. The UCI Institutional Review Board approved the experiment (HS# 2008–6432) and participants provided informed consent prior to the start of study procedures. For Stationary Mode, participants were asked to repeatedly and fully extend the arm rest with a forward reaching motion and then to pull it backward. For Overground Mode, participants were asked to propel the chair in a straight line for three meters (among other tasks), using their hemiparetic arm with Boost and their “good” arm to propel the other pushrim. They were given up to five trials to achieve this, where a trial was restarted if they turned off the line or repeated if they propelled for three meters but needed some assistance from the experimenter to keep the chair straight. To quantify the level of arm impairment for each participant, we used the Upper Extremity Fugl-Meyer Motor Assessment a widely used, valid scale that ranges from 0 (complete paralysis) to 66 (unimpaired arm movement) [36]. We quantified propulsion speed using video recordings, relying on the standardized floor tile sizes as distance markers. We also demonstrated Boost to 15 rehabilitation therapists (a mixture of occupational and physical therapists) at two hospitals and asked them to complete a survey about the device).

## III. Results

### A. Biomechanical Analysis

The kinematic performance of the wheelchair (for Boost and for a standard pushrim) is shown in Fig. 6. Note that the additional friction of the Boost drive system causes that system to have a greater constant deceleration.

**Fig. 7.**
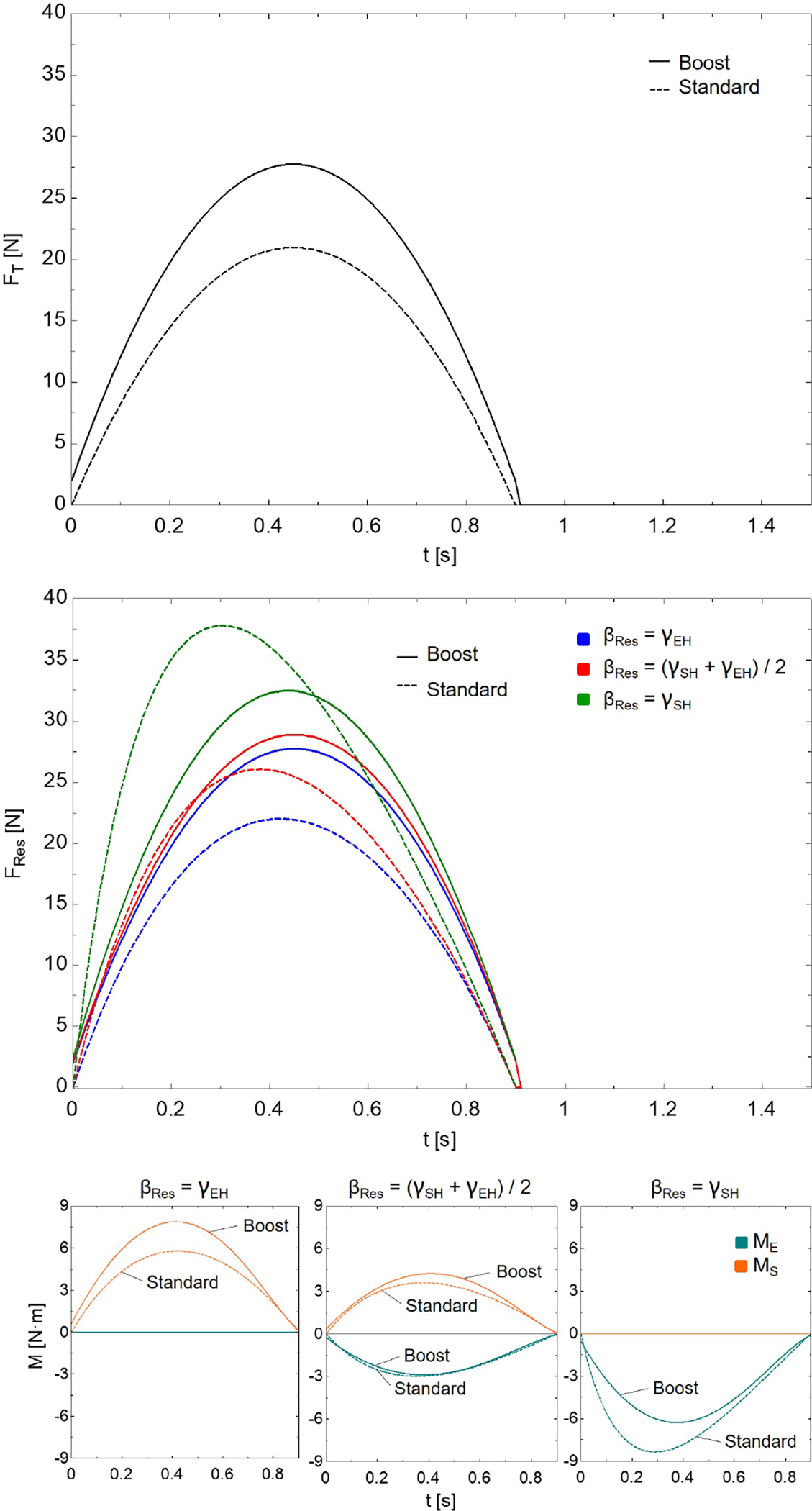
Top: Tangential force (ideal force) along the full cycle. Middle: Resultant force (applied force) for the three force application directions. Bottom: Elbow and shoulder torques along the full cycle for the three force application directions, which are defined in Fig. 5-c.

The ideal force (tangential force) required to propel the modelled Boost wheelchair is ~30% greater than the force required for the modelled pushrim propulsion (Fig. 7 top). Boost decelerates more quickly during the recovery phase due to the additional rolling friction produced by the drive system, thus requiring a higher force during the propulsion phase. Additionally, the resistance produced by the spiral spring must be overcome.

In contrast to the ideal force (tangential force), the magnitude of the applied force (resultant force) required to propel Boost ranges from −14% to +26% compared to the standard pushrim model, depending on the direction of force application (Fig. 7 middle). Likewise, the required joint torques vary between −25% (when the direction of the applied force is aligned with the shoulder joint and only elbow extension is required) to +36% (when the applied force is colinear with the forearm and only shoulder flexion is required) (Fig. 7 bottom). Finally, when the direction of the applied force is halfway between the elbow and shoulder directions, both a shoulder flexion torque and an elbow extension torque are required, with similar magnitudes when comparing the use of Boost and the standard wheelchair.

**Fig. 8.**
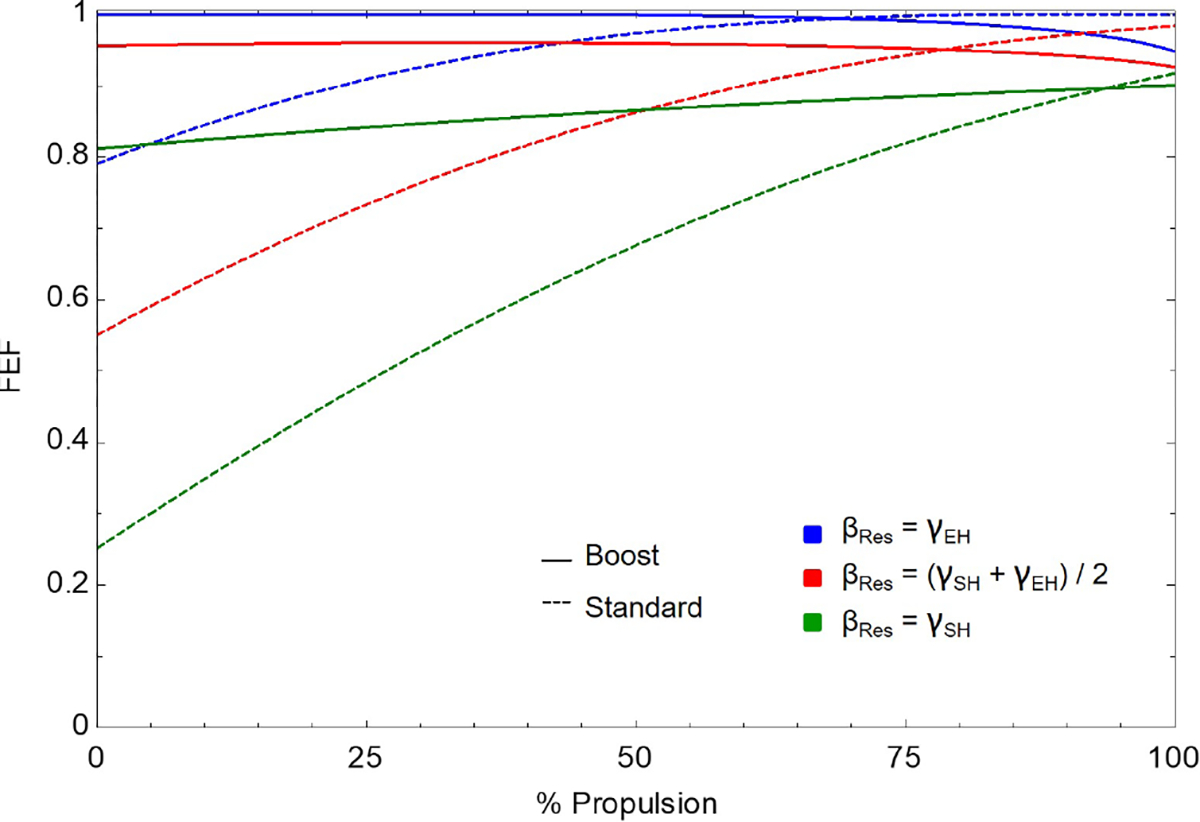
Fraction of effective force (FEF) along the propulsion phase for the three force application directions.

In terms of the fraction of effective force (FEF) (i.e. the ratio of the tangential force to the resultant force, a measure of propulsion efficiency), it remains steady and high (>0.8) for Boost along all the push phase for any of the applied force directions studied (Fig. 8). When using the standard pushrim technique, the FEF starts lower and increases through the propulsion phase for all applied force directions studied.

### B. Pilot Testing

**Fig. 9.**
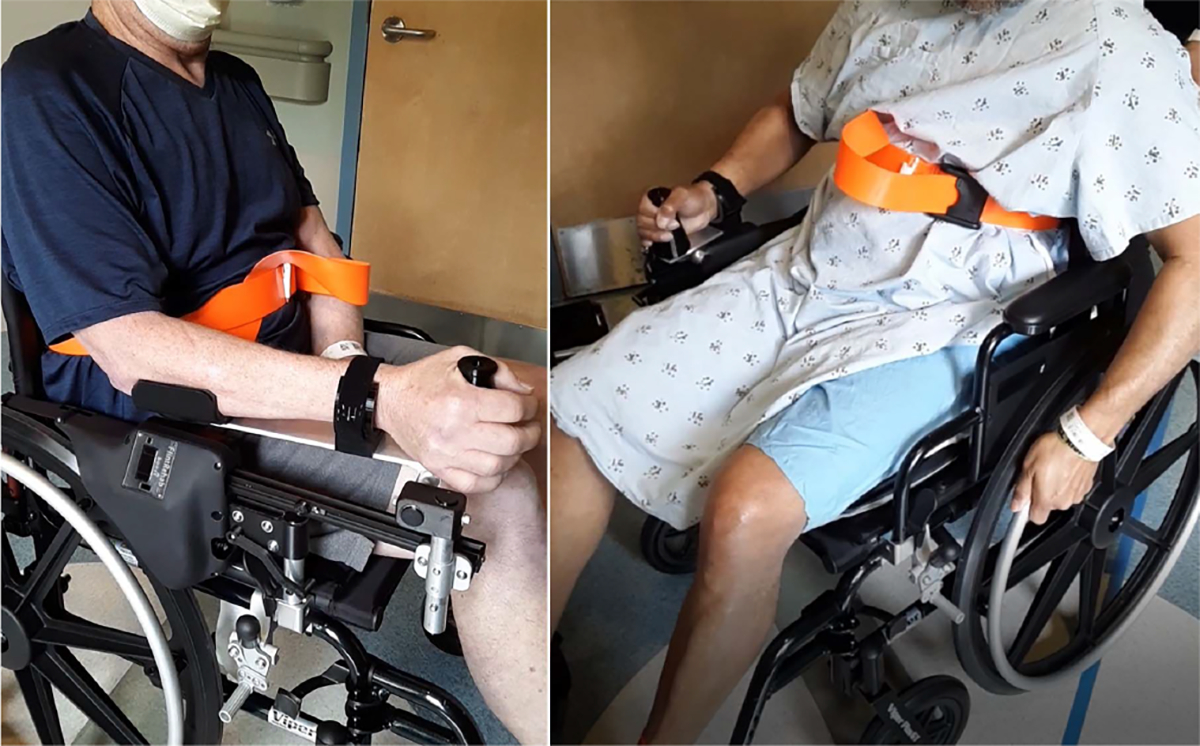
Left: Boost being used in Stationary Mode by Subject 2. Right: Boost being used in overground mode by Subject 4.

All five stroke inpatients were able to use Boost for arm exercise in Stationary Mode, moving the handle along the linear rail back and forth by actively extending their impaired arm (Fig. 9). Three participants (UEFM scores of 25, 35, and 58) were able to propel Boost overground in a straight path for three meters after practicing between 2 to 5 trials, achieving speeds of at least 0.2 m/s (Table I, see supplemental video). Two of these participants were not able to propel a manual wheelchair with the pushrim – i.e. Boost enabled bimanual propulsion when it was not possible previously. The two participants who could not propel Boost in Overground Mode had UEFM scores of 10. It is notable that they were still able to flex the shoulder and extend the elbow, then extend the shoulder and flex the elbow, to move the Boost handle forth and back in Stationary Mode.

The 16 physical and occupational therapists from two different hospitals who received a demonstration of Boost agreed that Boost was easy to set-up, intuitive for patients to use, may improve their patients’ motor recovery, and may improve their patients’ wheelchair mobility (Table II). In addition, the strong majority reported that they would use Boost during one-on-one therapy sessions, allow patients to use Boost in the clinic between therapy sessions, and would want patients to use Boost on their own at home. The exception was that only 38% recommended using Boost for unsupervised in-clinic use between therapy sessions if the patient was severely impaired.

## IV. Discussion

We described the design and preliminary testing of a novel wheelchair armrest developed to help exercise the hemiparetic arm outside of therapy sessions. We discuss first the relationship of Boost to previous wheelchair drive designs, followed by the biomechanical and experimental results, then directions for future research.

### A. Relationship to Previous Wheelchair Drive Designs

An interesting question is to what extent a dynamic, detachable, arm rest that enables wheelchair propulsion is related to previously proposed alternative wheelchair drive designs. [37] provided an extended review of different types of manual wheelchairs. While most of the wheelchairs included in this review used conventional pushrims, some also included mechanically geared wheels fitted to a standard manual wheelchair. Boost has a conceptual commonality with these geared pushrim approaches in that Boost also allows a potential means to change the transmission ratio by changing the reel diameter.

In addition to geared pushrims, several other alternative wheelchair drive designs have been proposed both in the scientific literature and in patent applications (e.g. [38], [39]). Many of these are crank-propelled designs [40], though the most common alternative design is the lever-drive [41], [42], with several options commercially available [43]. However, few previously proposed alternative designs are linearly actuated like Boost, where movements are guided parallel to the armrest (see, however [44]).

To our knowledge, most previously proposed alternative wheelchair drives are either built-in to the wheelchair or require complex/non-reversible installation. Such designs tend to be heavier, wider and/or longer, and less easy to fold than conventional pushrim wheelchairs. Boost appears unique in that it can be quickly attached and detached from a conventional wheelchair, maintaining much of the portability, size, and weight advantage of the pushrim base.

A non-powered solution was considered for this study. However, besides being obvious that full powered wheelchairs do not encourage arm exercise, it’s important to note that a combination of arm movement with a powered drive train, in a kind of hybrid system [45], [46], may be of interest for arm rehabilitation therapy, although it can add complexity, cost, and weight, among others.

Finally, some previous designs have implemented self-locking mechanisms to increase safety when ascending or descending long ramps [47]. Boost implements a similar feature for braking rearward motion using a spring-loaded brake, as described above.

### B. Biomechanical Analysis

The modelling results indicated that Boost requires a higher ideal propulsion force (tangential force) due to the additional friction and spring resistance of the drive system. However, it has a better force effectiveness, meaning that the applied force direction (across the range of likely applied force directions) is closer to the ideal force direction, due to the arm positioning and mechanism configuration, compared to a standard pushrim wheelchair. The pushrim FEF results from the model presented here are consistent with a study that found average FEF values between 0.26 – 0.81 when using a regular pushrim [35]. Whether Boost provides an advantage in terms of required joint torques will depend on the direction of the total force performed by the user, a question that should be addressed experimentally in future research and will likely depend on each user and, potentially, on training.

The biomechanical analysis results were generated based on specific assumptions, such as cycle times being appropriate for a stroke patient and the ground surface being flat and smooth. If any of these conditions changed in such a way as to increase the required force (for instance ambulating on a carpet or up a ramp, reducing propulsion time, etc.), the drive system resistance would have an increasingly minor effect and Boost performance (relative to the standard wheelchair) would increase. Further, as mentioned above, it is possible with Boost to modify the transmission ratio by changing the size of the reel, which would reduce the required joint torque, although it could affect motion patterns and cognitive performance.

### C. Pilot Testing

Boost’s low-friction, linear rail allowed even the most severely impaired patients we tested to exercise the arm in a forward/backward reaching motion. Repeating such a motion has been found to be therapeutic, helping to reduce arm long-term impairment after stroke in several studies [3], [18], [48]. Embedding such a therapeutic exercise right on the wheelchair may help improve accessibility to it. That is, Boost could enable patients with severe arm impairments to engage in large amounts of safe, beneficial arm exercise outside of one-on-one therapy sessions.

The two most severely impaired patients were not able to propel Boost in Overground mode. It is possible that such persons would eventually be able to take advantage of Overground mode for exercise by first practicing in Stationary mode to regain strength, and/or by waiting for arm recovery to progress further to the level needed for Overground mode.

Some of the patients who were able to propel the wheelchair using Boost were not able to with the standard pushrim propulsion method. The joint torque analysis showed that the required joint torques are not substantially different between Boost and the pushrim propulsion technique, for a range of feasible force application directions. So, what explains the difference? Boost guides arm movement so that the shoulder, elbow and wrist are roughly in the parasagittal plane, with shoulder flexion starting in neutral at the beginning of each push. We hypothesize that this posture is easier for stroke patients to achieve compared to abducting the arm and then extending the shoulder in order to grab the pushrim at the beginning of each push. Boost also reduces the effect of arm weight due to gravity by providing support to the forearm, which may make it easier for individuals to extend the elbow, because of abnormal coupling between shoulder abduction and elbow flexion [49]. Finally, Boost takes away the need to repetitively grasp and release the pushrim, which is a limitation for many people after stroke. These improved ergonomics likely contributed to the observation that some participants had success in propelling the wheelchair with Boost rather than a substantial change in mechanical advantage.

In terms of the therapists’ opinions, they were strongly positive about the potential for Boost to improve both arm recovery and overground mobility. Although we designed Boost based on the idea of providing a tool that is useful outside of therapy sessions, therapists also saw potential to use Boost during therapy sessions. Of note, they were hesitant to allow severely impaired patients to ambulate independently in the hospital, expressing safety concerns for this population, particularly due to the possibility of cognitive or attentional deficits early after stroke. Overground mode might therefore be most feasible for such patients when they are being transported with supervision to and from appointments within the hospital, such as returning from a therapy session to their room, or going to socialize in a common area, for example. These transition times could provide a novel, currently untapped, opportunity to achieve hundreds of additional rehabilitative arm movements if the patient self-propels using Boost.

Hemiplegic shoulder pain is one of the most common complications for individuals post stroke, occurring in up to 80% of individuals—most commonly in those who have little voluntary movement of their paretic limb [50]. It is important that Boost not aggravate this pain, and if possible, help prevent it. Of concern is the fact that high levels of conventional manual wheelchair use is associated with shoulder pain as well. In one longitudinal study [51] of individuals with paraplegia, 30% developed some shoulder pain within 36 months. Development of pain with pushrim propulsion was associated with shoulder abduction weakness and greater shoulder joint work during propulsion. On the other hand, there is evidence [52] that gentle arm exercise may reduce pain in the hemiplegic shoulder. Thus, by providing a means to gently exercise the arm, Boost may help reduce shoulder pain. Arm positioning also plays a role in hemiplegic shoulder pain [52], and we listened carefully to consulting therapists in designing the arm position that the patient uses to propel Boost. Therapists felt that the posture we settled on (arm in parasagittal plane, shoulder and elbow at neutral at push start) was optimal, compared especially to the shoulder-abducted and shoulder-extended arm posture needed to push a pushrim. This observation is supported by a recent large RCT that used a board-type arm support with a handle to achieve an arm position similar to Boost except with the arm held static [53]. The dynamic analysis presented here also indicates that the joint torques required with the arm in this improved posture are comparable to those required when the arm is in pushrim propulsion posture. Thus, we would expect less shoulder pain risk with Boost compared to pushrim propulsion (similar torques but better positioning). Ultimately this can only be verified by monitoring shoulder pain after extended use of Boost by a large group of users, an important direction for future research.

Determining the optimal dose and timing of arm movement exercise with Boost will be important. [54] showed that high dose constraint-induced therapy (3 hours per day) delivered starting about one week after stroke was less effective than low dose CI therapy (2 hours per day). This suggests that there exists an optimal dose of additional training. [55] then showed that task-specific training applied within the first month of stroke was slightly less effective at improving arm function than training applied at two to three months, although early training was better than no additional training as well as additional training applied in the chronic phase. This suggests that there exists an optimal timing window for providing additional training, although early training seems in general to produce better results than training conducted in the chronic phase.

### D. Future Directions

On the clinical side, Boost should be evaluated with a larger number of people with stroke to understand the size of the potential user population and for whom it is most appropriate. Further, a long-term training study should be conducted to quantify the effect of regular use of Boost on upper extremity recovery after a stroke.

On the design side, we are incorporating a linear position sensor, microcontroller, and small display into Boost to track the amount of arm movement the user achieves with Boost. Setting goals and providing feedback on arm activity may increase motivation and could provide a way for therapists to individualize exercise. Another interesting direction is to explore the effects of varying the reel and/or friction disk diameter, in order to alter the transmission ratio. Reducing the transmission ratio could allow even more severely impaired users to achieve overground ambulation.

## Supplementary Material

supp2-3187755

supp1-3187755

## Figures and Tables

**TABLE I:** Results of Testing Boost With Five Individuals Who Were Inpatients in an Acute Stroke Rehabilitation Unit

Subject	Upper Extremity Fugl-Meyer Score (out of 66)	Able to propel the wheelchair with the pushrim?	Able to use Boost in Stationary Mode?	Able to propel Boost in Overground Mode?	Overground Speed	# of trials required to learn to propel Boost overground

1	25	No	Yes	Yes	0.23	2
2	10	No	Yes	No	-	-
3	58	No ^a^	Yes	Yes	0.21	2
4	35	Yes	Yes	Yes	0.2	5
5	10	No	Yes	No	-	-

a Due to grip weakness

**TABLE II:** Results From Survey of 15 Physical and Occupational Therapists

Statement	Stationary Mode	Overground Mode
Boost is easy to set-up (1 = Strongly Disagree, 5 = Strongly Agree)	4.4	4.2
Boost is intuitive for patients to use	4.2	4.0
Boost may improve patients’ motor recovery	4.4	4.3
Boost may improve patients’ wheelchair mobility	4.6	4.6
	Moderately Impaired Individuals	Severely Impaired Individuals
Would you recommend Boost during one-on-one therapy?	100%	88%
.. for unsupervised in-clinic use between therapv sessions?	88%	38%
... for patients to use on their own at home?	94%	74%
